# Pseudo-outbreak of Pseudomonas aeruginosa: a case series and investigation of cross-contamination in the microbiology laboratory

**DOI:** 10.1017/ash.2025.369

**Published:** 2025-09-24

**Authors:** Shinya Hasegawa, Poorani Sekar, Bradley Ford, Katie Halliwill, Karen Brust

**Affiliations:** 1 University of Iowa Health Care; 2 University of Iowa; 3 Univesity of Iowa Hospitals and Clinics; 4 UIHC

## Abstract

**Background:** Distinguishing outbreaks from pseudo-outbreaks is essential in healthcare settings. Pseudo-outbreaks are defined by an increase in identified organisms without clinical evidence of infection. Here we report two cases involving Pseudomonas aeruginosa identified in clinical specimens, later determined to represent a pseudo-outbreak. **Methods:** Patient #1 had vertebral osteomyelitis and epidural abscess; intraoperative and blood cultures grew Streptococcus mitis/oralis. Four days post-surgery, one colony of P. aeruginosa grew from one of three intraoperative aerobic cultures. Patient #2 developed a fracture-related infection of the ankle and underwent debridement and hardware removal; all intraoperative cultures grew methicillin-susceptible Staphylococcus aureus. Four days later, two colonies of P. aeruginosa were detected in one of three intraoperative aerobic cultures. Both these findings were deemed unusual, leading to an outbreak investigation, including chart review and laboratory investigations, to identify a source of contamination. **Results:** The two cultures were received and set up one day apart by different staff. Subsequently, the WASPLab incubation system’s photographic record of the plates demonstrated no P. aeruginosa within the expected first 48 hours, suggesting contamination during culture collection or processing was unlikely. Further review revealed a heavily inoculated culture of P. aeruginosa was processed by the same laboratory technician on an open bench immediately before handling plates for patients #1 and #2. P. aeruginosa typically grows within 24 hours of incubation, and the colony morphology of the contaminated plates matched those of the heavily inoculated culture. Furthermore, both patients had monomicrobial growth of a likely pathogen causing their infection. Therefore, we concluded that this was cross-contamination, likely via aerosolization or improper plate handling. For patient #1, cefepime was discontinued on post-operative day six and switched to ceftriaxone, completed for six weeks, followed by suppressive therapy with amoxicillin, with no recurrence at three months. Patient #2 completed six weeks of cefazolin without anti-pseudomonal coverage, also without recurrence at three months. **Conclusions:** The pseudo-outbreak likely stemmed from cross-contamination caused by aerosolization or handling heavily inoculated P. aeruginosa cultures near the time and location of the two patients’ plates on an open bench. Awareness of such rare contamination pathways is critical for microbiology labs and clinicians, especially when handling hazardous isolates such as Brucella spp. Careful record keeping and digital storage of serial plate images can narrow the source of contamination, and active surveillance by trained epidemiology personnel is essential to detecting pseudo-outbreaks. Clinical and microbiological correlation should guide treatment to avoid unnecessary antibiotic treatment.

